# Tracking and interpreting long-range chromatin interactions with super-resolution live-cell imaging

**DOI:** 10.1016/j.ceb.2020.11.002

**Published:** 2020-12-09

**Authors:** Hugo B. Brandão, Michele Gabriele, Anders S. Hansen

**Affiliations:** 1 Department of Biological Engineering, Massachusetts Institute of Technology, Cambridge, MA, 02139, USA; 2 Graduate Program in Biophysics, Harvard University, Cambridge, MA, 02138, USA

**Keywords:** Enhancers, Chromatin looping, Super-resolution live-cell imaging, SRLCI, CTCF, Cohesin, Gene regulation, Microscopy, 3D genome, Dynamics

## Abstract

Mammalian genomes are organized and regulated through long-range chromatin interactions. Structural loops formed by CCCTC-binding factor (CTCF) and cohesin fold the genome into domains, while enhancers interact with promoters across vast genomic distances to regulate gene expression. Although genomics and fixed-cell imaging approaches help illuminate many aspects of chromatin interactions, temporal information is usually lost. Here, we discuss how 3D super-resolution live-cell imaging (SRLCI) can resolve open questions on the dynamic formation and dissolution of chromatin interactions. We discuss SRLCI experimental design, implementation strategies, and data interpretation and highlight associated pitfalls. We conclude that, while technically demanding, SRLCI approaches will likely emerge as a critical tool to dynamically probe 3D genome structure and function and to study enhancer–promoter interactions and chromatin looping.

## Introduction

The expression of specific genes only in specific cell types and at precise developmental stages is achieved through exquisite regulation of gene expression. In metazoa, genomic regions known as enhancers are key units of gene expression regulation [1,2]. Because enhancers can regulate gene expression across vast genomic distances of hundreds of kilobases to megabases, understanding long-range chromatin interactions (looping) is essential to understanding gene regulation.

It is well established that chromatin looping plays a central role in gene expression and disease. Germline mutations to chromatin looping regulators frequently result in developmental disorders [3–5], while somatic mutations to looping regulators are often found in cancer [6–8]. Moreover, specific disruption of individual chromatin loops can cause human developmental defects [9] and activation of oncogenes [10,11].

We can distinguish at least two major classes of long-range chromatin interactions: structural loops and enhancer–promoter (E–P) interactions. Structural loops fold the genome into domains [12] and are believed to regulate gene expression indirectly by promoting or preventing the formation of E–P interactions [13]. In contrast, E–P interactions directly regulate gene expression, although the mechanisms remain unclear [1,14,15].

Structural loops and E–P interactions may be formed by different physical principles [16]. While many aspects of structural loop dynamics remain unknown, mounting evidence suggests that many structural chromatin loops are formed by DNA loop extrusion involving cohesin complexes and CCCTC-binding factor, CTCF [17–19]. By contrast, how functional E–P interactions are formed, maintained, exert their function, and dissolve remains less clear, although cohesin and loop extrusion likely also play a role [20,21].

**Figure 1:**
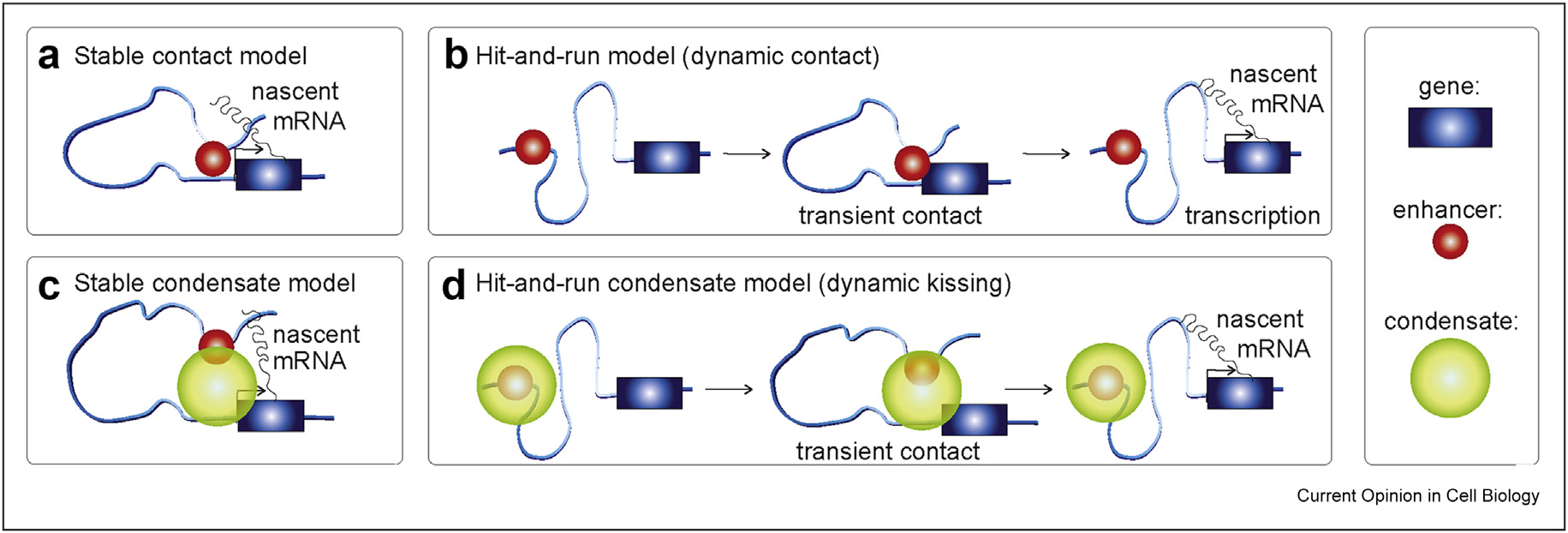
Models for enhancer–promoter (E–P) interactions: **(a)** Stable contact or stable loop model. **(b)** Dynamic contact model (hit-and-run). **(c)** Stable condensate model. **(d)** Dynamic condensate model (hit-and-run or dynamic kissing). Black arrow at the gene indicates active transcription.

Despite our limited understanding of E–P interactions, we can categorize current E–P models into four broad classes (Fig. 1). First, the classic E–P model is the stable contact model (Fig. 1a). In this model, a stable E–P loop brings transcriptional activators bound at the enhancer into contact with the gene promoter, facilitating RNA Pol 2 recruitment and induction of gene expression [14,15]. Evidence for this model comes from the demonstration that forcing E–P looping is sufficient to activate β-globin expression [22,23]. However, a study that simultaneously visualized DNA and RNA by fluorescence *in situ* hybridization (FISH) in *Drosophila* found only a small correlation between E–P proximity and gene expression for the three genes studied [24]. Furthermore, a recent study found that E–P distance increases during gene activation, which is the opposite of what the stable contact model would predict [25]. Rather than a stable contact model, these observations would be consistent with a second ‘hit-and-run’–type contact model: the dynamic contact model (Fig. 1b). If there is a delay between E–P contact and transcription initiation, by the time the nascent mRNA appears, the enhancer may no longer be in contact with the gene [26].

Beyond contact-type models, recent studies have found that the transcriptional machinery – including transcription factors, Mediator, BRD4, CDK9, and RNA Pol 2 itself – can form higher-order clusters and perhaps even liquid–liquid phase-separated condensates [27–30] leading to condensate models (Fig. 1c, d). Condensate models can, in principle, explain why direct E–P contact is only weakly correlated with transcription [24,25,31]: because condensates are reported to be quite large (~200–500 nm), they could form a ‘bridge’ from the enhancer to the promoter, thereby alleviating the need for direct contact [25,31]. Both stable and dynamic versions of the condensate model have been proposed (Fig. 1c, d).

The disagreements regarding the role of 3D genome organization in regulating gene expression and our poor mechanistic understanding of E–P interactions [32] are partly due to methodological limitations. Specifically, most methods used to disentangle the functional role of the 3D genome are snapshot methods such as Hi-C, DNA-FISH, or single-cell genomics. Snapshot approaches are blind to time and dynamics [1,33–35] and susceptible to chemical fixation artifacts [36]. While these methods are powerful, the loss of dynamical information is a significant limitation. Distinguishing stable and dynamic models of E–P interactions (Fig. 1) requires an ability to follow E–P interactions in living cells over time. It thus cannot be achieved with snapshot methods. Similarly, distinguishing E–P contact (~<50 nm) from E–P proximity or condensate models (~200–500 nm) requires very high spatial resolution, which calls for methods with spatial resolutions of ~30 nm or better.

Simultaneous high temporal and high spatial resolution can in principle be achieved with 3D super-resolution live-cell imaging (SRLCI). SRLCI is thus ideally suited to probing long-range chromatin interactions, including both structural loops and E–P interactions. For example, using SRLCI, it is possible to dynamically follow both E–P communication and nascent transcription in the same living cell for extended periods as required to distinguish static from dynamic models of E–P interactions as recently demonstrated in *Drosophila* embryos and mouse embryonic stem cells [37,38]. However, a downside of SRLCI is its complexity, including experimental design, choice of locus, genome editing, locus labeling strategy, appropriate microscope calibration, image analysis, and biophysical analysis of locus trajectories.

Indeed, two recent SRLCI studies came to opposite conclusions for the mechanisms of E–P interactions, with one reporting clear evidence of E–P looping mediating gene activation [38] and the other reporting no role for E–P proximity in gene activation [37]. These differences could be due to distinct locus-specific mechanisms (Fig. 1) or due to differences in experimental design. Here, we therefore provide a brief discussion of SRLCI to study long-range chromatin interactions in general and aim to illuminate both the design and interpretation of SRLCI approaches.

## Main text

### Choice of the biological system and experimental design

**Figure 2:**
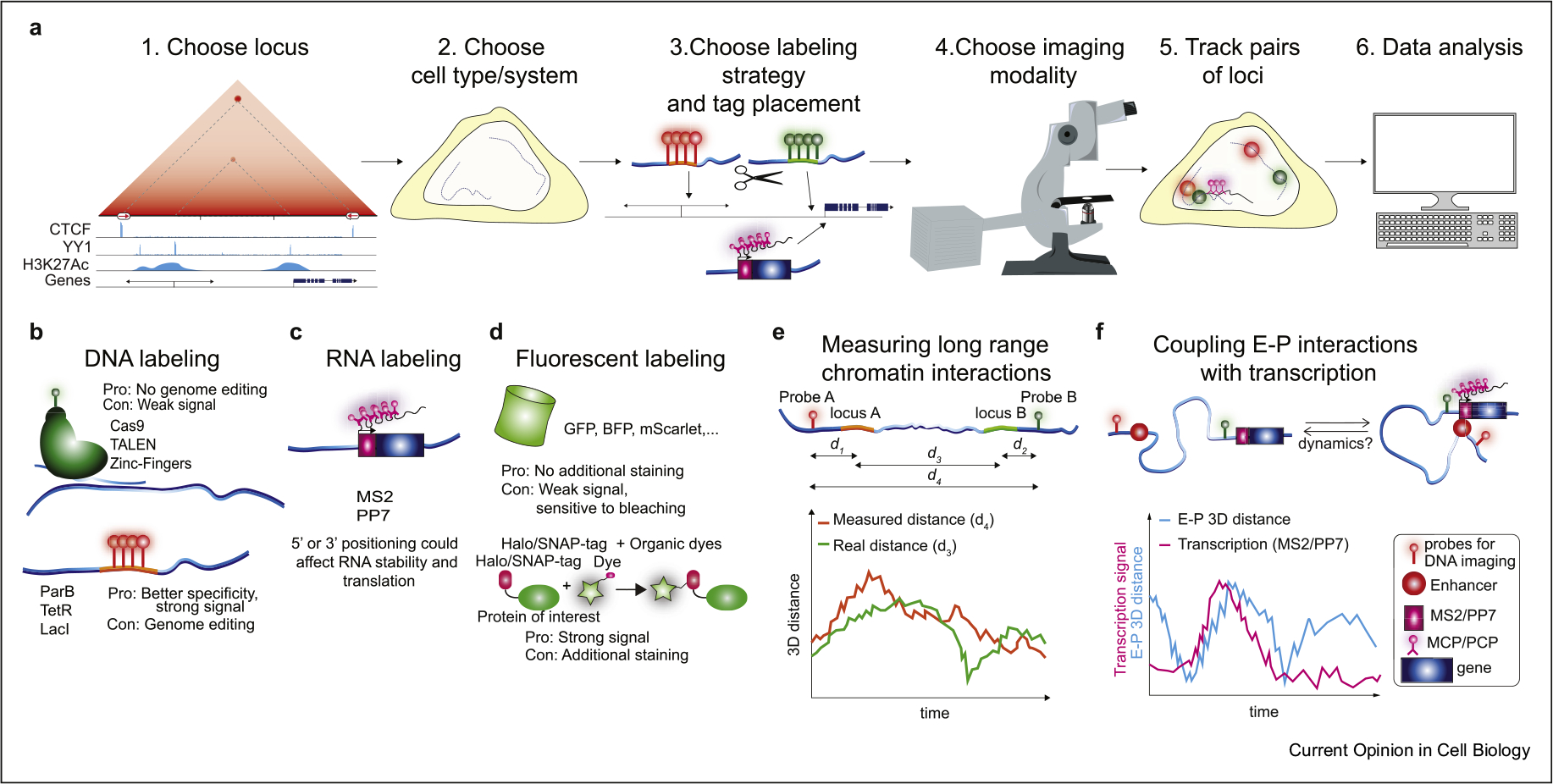
Workflow of SRLCI experiments, choice of biological system, and experimental design. **(a)** Workflow diagram for SRLCI design, experiments, and analysis. **(b)** Methods for fluorescent DNA labeling: upper methodologies do not require genome engineering. The lower ones require genome engineering but may provide a higher signal-to-noise ratio. **(c)** RNA labeling is achieved through engineering with MS2 or PP7. 5ʹ-tagging provides better time resolution than 3ʹ-tagging at a higher risk of interfering with translation. **(d)** Fluorescent labeling can be accomplished with fluorescent proteins fused with the protein of interest or by fusion of Halo/SNAP-tag systems in combination with organic dyes, which provide a stronger signal. **(e)** Sketch of loci of interest, their distance, and the tether length between loci of interests and fluorescent labels. **(f)** Sketch of the putative relationship between 3D E–P distance and detected nascent transcription. SRLCI, super-resolution live-cell imaging.

Given the limitations inherent to SRLCI approaches, a well-designed strategy is required. In this section, we briefly discuss experimental design considerations related to locus choice, cell type, and locus labeling strategy (Fig. 2a).

**Figure 3:**
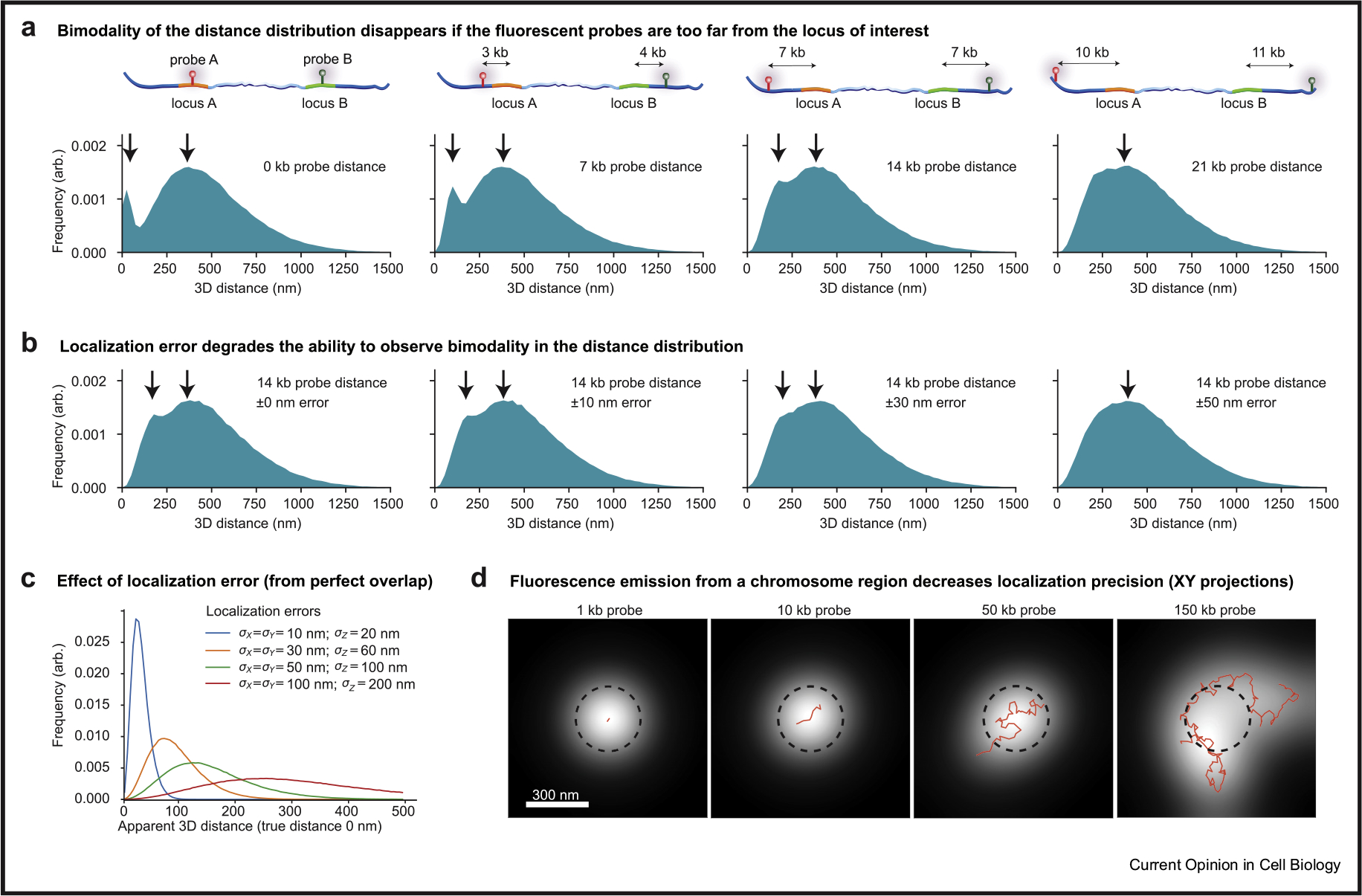
Fluorescent labeling design pitfalls. We generated 3D polymer simulations of a 525 kb chromatin interaction where contacts between monomers (defined as <50 nm distances) occurred between locus A and B approximately 5% of the time. The simulations are used to demonstrate the following: **(a)** the effect of fluorescent label placements with respect to loci of interest (*d*_1_+*d*_2_ in Fig. 2e) and the resulting histogram of measured distances (bottom row); **(b)** the effect of localization error on the ensemble of measured 3D distances (with Gaussian-distributed errors generated for each dimension using the standard deviations indicated in the panels); **(c)** the effect of localization error (conditioned on the case of perfect overlap; i.e. true distance is 0 nm); and **(d)** the effect of fluorescently tagging a chromatin region with finite size probes (the resulting fluorescence emission is the white spot, the simulated chromatin is superimposed in red, and the dashed black circle is to illustrate the true PSF width and to help visualize the fluorescent emission asymmetry and broadening). PSF, point spread function.

First, after formulating a clear biological question, the abundance of available genomics data, such as Hi-C, Micro-C, ChIP-Seq, RNA-Seq, and ATAC-Seq, can be used to identify loci of interest [35]. Interactions that appear prominently in contact-type data (e.g. Hi-C, Micro-C) are also likely to occur frequently in single cells making such loci good candidates for SRLCI (Fig. 2a–1). For example, DNA-FISH measurements in human fibroblasts suggest that the strongest interactions seen in Hi-C data correspond to colocalizations within ~150 nm in ~20% of cells [39]. Furthermore, if the aim is to distinguish between competing mechanisms of E–P interactions (Fig. 1), then choosing a gene regulated by a single enhancer will generally be preferable compared with a gene regulated by several redundant enhancers. Matching locus choice and cell type choice is essential (Fig. 2a–2). The cell type should be appropriate for the biological question and amenable to imaging (ideally adherent and low cell movement) and genome editing. Finally, one must choose a fluorescent tagging approach (Fig. 2a–3). A full review of labeling strategies is beyond the scope of this review, and we refer the reader to other excellent reviews on this topic [40,41]. Briefly, the most popular strategies for DNA labeling use either exogenously expressed dCas9 [42] or endogenously inserted arrays of binding sites to be bound by ‘conventional’ DNA-binding proteins such as LacI, TetR, TALENs, and zinc fingers [43] or by ‘multimerizing’ DNA-binding proteins such as ParB [44] (Fig. 2b). dCas9 labeling conveniently avoids genome editing. However, except for in repetitive regions [45], the reported signal-to-noise ratio for dCas9 imaging is generally far below what can be achieved with endogenously inserted arrays [46]. Relatedly, nascent RNA can be visualized using MS2/PP7 [40]. Inserting MS2/PP7 hairpins into either the 5ʹ- or 3ʹ-UTR of a gene allows visualization of the nascent mRNA by coexpression of a fluorescently tagged MCP/PCP hairpin binding protein (Fig. 2c).

For fluorescent labeling of DNA- and RNA-binding proteins, one can use fluorescent proteins such as GFP or self-labeling tags such as Halo-Tag or SNAP-Tag (Fig. 2d). While fluorescent proteins are smaller and simpler, self-labeling tags can be conjugated with far superior organic dyes such as Janelia Fluor dyes [47].

When designing a labeling strategy (Fig. 2e), a fundamental trade-off is the distance from the locus of interest (e.g., enhancer, CTCF site) and the fluorescent label: a short distance provides a better reporter but increases the risk of disrupting endogenous regulation. Importantly, however, the ability to discriminate between “physical proximity”‘ and “physical contact”‘ E–P models (Fig. 1) depends intimately on the distance of the fluorescent tag from the genomic object of interest (Fig. 3a).

To illustrate this point, we carried out 3D polymer simulations of chromatin (refer Supplementary Information; [48,49]) to test how the fluorescent tagging approach affects the observed 3D distance distributions between the two tagged chromosomal loci. We simulated a CTCF loop domain, which is formed through cohesin-mediated loop extrusion, and where the two CTCF anchors are in a looped configuration for an average of 5% of the time. In the simulations, if the fluorescent probe is placed directly on top of the locus of interest (i.e. the CTCF sites), then a clear bimodal distribution of distances emerges (Fig. 3a, left panel; as indicated by the two arrows). However, if the distance between the fluorescent tag and the CTCF sites is modestly increased (e.g. up to 10 kb away from each CTCF site), the bimodality of the distribution disappears (Fig. 3a). Thus, although a CTCF loop is present 5% of the time, it is not possible to see the expected bimodality in the 3D distance histograms. In real SRLCI experiments, localization error/uncertainty of tens of nanometers is inevitable. Modest localization uncertainties further corrupt the ability to see the bimodality of the distribution (Fig. 3b). Indeed, even in the case where there is perfect overlap (true distance of 0 nm) between two loci, one can measure apparent 3D distances of more than 200 nm due to localization uncertainty (Fig. 3c). Thus, even with modest localization errors, contact-type E–P interactions (<50 nm) can yield apparent measurements of E–P 3D distances of ~150–300 nm. Altogether, these factors arising from localization error and the distance of the fluorescent probe to the looped locus may make it challenging to distinguish between ‘contact-type’ from ‘proximitytype’ interactions. If not carefully considered, this could lead one to draw incorrect mechanistic conclusions.

A careful experimental design is also required for imaging nascent RNA using MS2/PP7 approaches (Fig. 2f). Here, one key consideration is 5ʼ- vs. 3ʼ-UTR tagging: tagging the 5ʼ-UTR is much more likely to disrupt transcription and/or translation, whereas 3ʹ-UTR tagging avoids this at the cost of making a poor transcriptional reporter. Specifically, for all but the shortest genes, 3ʹ-UTR tagged genes will only show an MS2/PP7 signal long after transcription initiation took place. For example, for a 10 kb gene and transcription rates of ~10–60 bp/s, by the time the MS2/PP7 hairpins are transcribed and thus visible, between ~3 and 17 min will have elapsed from the moment the gene had begun transcribing. Coupling this temporal uncertainty with the spatial uncertainty arising from the proximity of DNA tags to their targets may make it highly challenging to identify ‘hit-and-run’ interactions such as those in the dynamic E–P models (Fig. 1).

### Microscope modality and acquisition optimization

**Figure 4:**
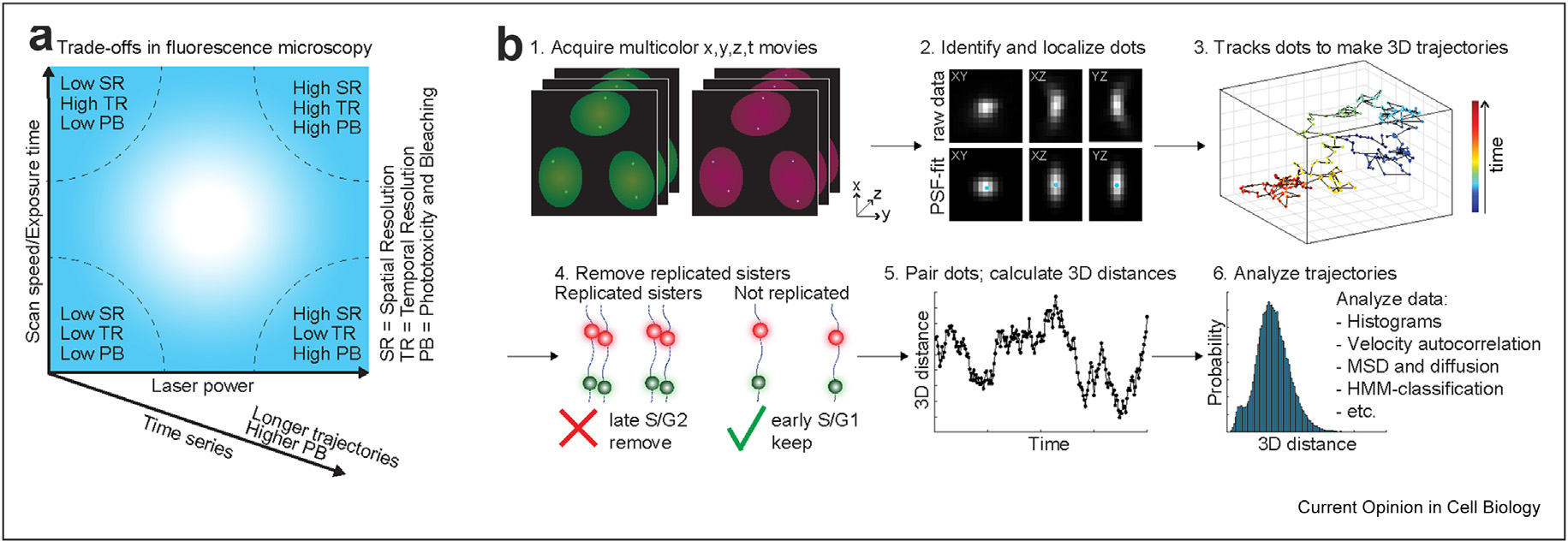
Microscopy overview and particle tracking considerations. **(a)** Overview of trade-offs in fluorescence microscopy. High laser power provides a high signal, high spatial resolution, and low localization error but leads to phototoxicity and bleaching. Fast scan speed/exposure time yields high temporal resolution and limits ‘motion blurring’ but at the cost of lower signal and lower spatial resolution. Simultaneous use of high laser power and fast scan speed/exposure time can yield both high spatial and high temporal resolution but at the cost of high phototoxicity and bleaching. Time-lapse imaging makes it possible to follow the same cell and loci over time, detect dynamics, and record trajectories. However, longer time series lead to higher photobleaching. Optimal SRLCI imaging requires careful parameter optimization according to these considerations. **(b)** Workflow diagram for image analysis, trajectory generation, and trajectory analysis. SRLCI, super-resolution live-cell imaging.

After setting up a biological system, the next step is choosing the imaging modality and optimizing acquisition conditions (Fig. 2a). Generally speaking, one faces a trade-off between temporal resolution, spatial resolution, and imaging duration (typically limited by photobleaching and phototoxicity) (Fig. 4a). For example, increasing laser power and exposure time increases the signal-to-noise ratio and spatial resolution at the cost of higher photobleaching rates and lower temporal resolution [50,51]. Thus, designing an optimal SRLCI experiment involves careful optimization of acquisition parameters to find the best compromise between temporal resolution, spatial resolution, and imaging duration (Fig. 4a).

While a full discussion of microscope modalities is beyond the scope of this review and can be found elsewhere [50,51], we will briefly comment on some key considerations. SRLCI is akin to single-molecule localization microscopy techniques such as STORM, PALM, and SPT. It achieves ~10–40 nm localization precision by inferring the centroid XYZ-coordinates of a ‘dot’ corresponding to the fluorescently labeled locus of interest. Single-molecule localization microscopy localization algorithms generally assume this dot to be a diffraction-limited point source. However, this assumption may not hold if a fluorescent array covering many kilobases is used. For instance, in simulations of a chromatin locus, where varying lengths of DNA are fluorescently tagged (Fig. 3d), we find that the underlying chromatin conformation can influence the observed ‘dot’. For typical DNA-FISH probe sizes of ~100–200 kb and even probes as small as 10 kb, we can observe broadening of the fluorescence signal and asymmetric dots (Fig. 3d), which result in higher localization uncertainties. Thus, choosing a smaller label is desirable.

Because the precision of localization microscopy improves proportionally to the square root of detected photons (√N), achieving a good signal-to-background is required for precise localization [52]. Thus, to improve localization precision, one should choose a detector (e.g., camera or GaAsP PMT) with high quantum efficiency and low noise. Similarly, because emission photon detection is proportional to NA^2^, one should choose a high numerical aperture objective (NA~>1.4). Finally, the magnification should be chosen such that the pixel size of the detector approximately matches the width of the point spread function (PSF) of the object of interest [52,53].

The three most frequently used imaging modalities are widefield, confocal, and light-sheet microscopy [50,54]. Widefield microscopy is straightforward and fast but suffers high background because it does not optically section the sample. Confocal microscopy achieves excellent optical sectioning but at the cost of high photobleaching and slower acquisition speeds. In contrast, light-sheet microscopy can simultaneously achieve excellent optical sectioning and high acquisition speeds but at the cost of smaller fields of view, increased instrument complexity, and lower photon detection efficiency. Finally, because living cells may move significantly, achieving high acquisition speed can help minimize ‘motion blurring’ of loci [55,56], which otherwise further degrades localization precision.

An additional challenge in multicolor SRLCI is correcting chromatic shifts [57]. Two perfectly colocalizing loci labeled in two different colors will nevertheless appear offset due to unavoidable chromatic aberrations. Chromatic shifts can be many tens of nm in each dimension and are typically measured using very small multicolor fluorescent beads. The shift can change significantly across the field of view in both X, Y, and Z and can vary from day to day and sample to sample. Thus, meticulous measurement and correction of chromatic shifts in XYZ are required to measure 3D distances accurately and infer chromatin interactions.

### Computational image and data analysis

4D SRLCI movie acquisition (z stacks of XY images over time) is followed by data analysis (Fig. 4b–1). The first step is to identify and precisely localize dots. Traditionally, multi-color 3D z-stacks are read-in, filtered (using e.g., a Difference of Gaussian, Laplacian of Gaussian filter, or wavelet methods) and dots are identified by thresholding [50,51,58]. More recently, machine learning approaches have also emerged as powerful extensions of traditional image processing [59]. Regardless of how dots are identified, the next step is extracting the locus X, Y, Z coordinates with nanometer precision – this is the step that brings ‘super-resolution’ to localization microscopy approaches (Fig. 4b–2). The PSF describes how emission from a point source (e.g. a single fluorescent molecule) will be ‘blurred’ when viewed in the microscope. By fitting the observed ‘blur’ (PSF fitting), the PSF centroid coordinates can be obtained with nanometer precision [52,53]. Alternatively, an intensity-weighted centroid estimation can be more robust than PSF fitting if cell movement or locus diffusion is comparable with the acquisition speed [56].

Once loci have been identified and precisely localized in each frame, the next step is to track them across time to form trajectories (Fig. 4b–3) [58,60,61]. Given the low particle density (~two pairs of loci per nucleus per frame, in case of homozygous targeting) and that chromatin moves slowly, this step is relatively straightforward. Next, replicated loci must be removed. Since the ‘average cell’ is approximately halfway through the cell cycle, approximately half of all dots will have replicated (Fig. 4b–4). After replication, the two dots on sister chromatids can ‘breathe’ and appear as both ‘singlets’ and ‘doublets’ over time [37,62]. Because accurate localization of replicated loci is generally not possible, it is essential that trajectories with replicated loci are removed and not further analyzed. Finally, trajectories (X, Y, Z, t) of single loci must be paired with the corresponding trajectory in the other color on the same chromosome (Fig. 4b–5). This can conveniently be achieved by minimization of the sum of frame-by-frame distances.

After the generation of paired trajectories, the trajectories can be analyzed to address the motivating biological question (Fig. 1b). For example, if E–P imaging is paired with nascent RNA imaging, the temporal correlation between E–P proximity and nascent RNA signal can be calculated to evaluate stable vs. dynamic E–P interaction models (Fig. 1) [37,38]. Although temporal information is lost, the simplest analysis is to compare histograms of 3D distances (Fig. 4b–6). This can be powerful when combined with biological controls: Having observed a bimodal histogram of 3D E–P distances, Chen et al. [38] compared different *Drosophila* lines with and without a functional enhancer to demonstrate that the histogram peak at short distances corresponded to functional E–P interactions. Similarly, to generate a “positive control”‘ for E–P proximity, Alexander et al. [37] deleted the ~111 kb that separates the *Sox2* enhancer and promoter.

Methods incorporating time domain information include mean squared displacement and velocity autocorrelation analysis, which can be combined with biophysical and polymer modeling [63] to distinguish between mechanistic models that regulate the timing of pair-wise long-range chromatin interactions [64]. Similarly, statistical inference methods can be combined with kinetic models to describe changes to E–P interaction states (‘unpaired’, ‘paired and gene OFF’, ‘paired and gene ON’) [38]. Indeed, polymer physics-based modeling accounting for multipoint correlations can help uncover additional information including how stress propagates over different genomic distances [65], help define macroscopic observables such as the effective confinement radius between two points [66], or infer the degree of cross-linking of the chromosome [67]. These recent studies highlight the importance of measuring the temporal dynamics between chromosomal loci. Future work will need to be done to test these various models of chromosome organization.

While the aforementioned studies have blazed a trail for pair-wise tracking of chromatin interactions, this field is only now emerging. There is thus a great need to benchmark and critically evaluate analysis methods going forward. We suggest that comparisons to realistic polymer simulations, where the ground truth is known, can provide a powerful means of achieving this.

### Limitations of SRLCI

Although powerful, SRLCI approaches also have their disadvantages. First, when choosing what to label, one must generally have an idea of ‘what to look for’, which is not required for unbiased genomics approaches. Second, due to overlap in fluorescence excitation/emission, one is typically limited to four or fewer distinct fluorescent labels (e.g. DNA loci, nascent RNA, protein). Third, the throughput of SRLCI approaches is limited, both due to the extensive genome editing required to tag endogenous loci, which limits the number of chromatin interactions that can be studied, and due to the high-resolution microscopy required, which typically limits the number of single cells that can be studied. Fourth, very careful design is required. For example, one must carefully balance between placing the fluorescent labels (Fig. 2) sufficiently close to the locus of interest to provide a reliable reporter (Fig. 3a and b), yet sufficiently distant to avoid disrupting endogenous regulation. Fifth, the technical complexity is high and requires extensive biological, genome editing, microscopy, and computational expertise, as well as careful attention to biases. For example, when attempting to distinguish contact from proximity models, too large a tether length between loci of interest and their fluorescent label (Fig. 3a), inadequate correction for chromatic shifts, and/or too high localization error (Fig. 3b and c) may cause contact interactions (~<50 nm) to inadvertently appear as much longer distances (~>150 nm). Sixth, data interpretation is challenging. Further development of computational methods to interpret dynamic loci trajectories will be required. We expect continued refinement of SRLCI methodology over the coming years to address many of these limitations.

## Conclusions

Genomics methods excel at unbiased profiling of whole genomes. However, despite intense research efforts, the locus-specific mechanisms of E–P interactions (Fig. 1) and the dynamics and function of 3D genome organization and chromatin looping remain unclear. While still highly technically challenging to implement and interpret, SRLCI can yield mechanistic insight into these processes that are otherwise unobtainable with static genomics and fixed-cell imaging approaches that are blind to time. Overall, with continued technical refinement, we believe that SRLCI will emerge as a powerful tool to disentangle the 3D genome and long-range chromatin interactions.
